# Platelet Jak2 deficiency accelerates atherosclerosis with increased inflammatory response

**DOI:** 10.1016/j.jbc.2025.110603

**Published:** 2025-08-16

**Authors:** Daeun Kim, Yu Zhe Li, Jiaqi Yang, Brian Lin, Meng Yun Wang, Jessie Jia Yu Liu, Jia Qi Adam Bai, Gedaliah Farber, Faisal Abdul Razzaq, Evan Pollock-Tahiri, Christopher Khoury, Xi Lei, Guangheng Zhu, Kay-Uwe Wagner, Anastasia A. Tikhonova, Heyu Ni, Minna Woo

**Affiliations:** 1 Toronto General Hospital Research Institute, University Health Network, Toronto, Ontario, Canada; 2 Institute of Medical Science and Department of Pharmacology and Toxicology, University of Toronto, Toronto, Ontario, Canada; 3 Princess Margaret Cancer Centre, University Health Network, Toronto, Ontario, Canada; 4 Department of Immunology, University of Toronto, Toronto, Ontario, Canada; 5 Department of Laboratory Medicine, Keenan Research Centre for Biomedical Science, St Michael's Hospital, Toronto, Ontario, Canada; 6 Department of Oncology, Wayne State University School of Medicine and Tumor Biology Program, Barbara Ann Karmanos Cancer Institute, Detroit, Michigan, USA; 7 Department of Laboratory Medicine and Pathobiology, University of Toronto, Toronto, Ontario, Canada; 8 Canadian Blood Services Centre for Innovation, Toronto, Ontario, Canada; 9 Division of Endocrinology and Metabolism, Department of Medicine, University Health Network/Sinai Health System, University of Toronto, Toronto, Ontario, Canada

**Keywords:** Janus kinase (JAK), atherosclerosis, platelet, bone marrow, inflammation

## Abstract

Cardiovascular disease (CVD) is the leading cause of mortality worldwide, of which atherosclerosis is the major pathology. Chronic inflammation underlies atherosclerosis, and Janus kinase 2 (Jak2) is a critical signaling node that mediates this process. *Jak2*^*V617F*^ activating mutation has recently been implicated in clonal hematopoiesis of indeterminate potential as an emerging major CVD risk factor. While platelets’ role in hemostasis and thrombosis is well-established in CVD, the essential role of platelet Jak2 in mediating inflammation in atherosclerosis is unknown. To this end, we assessed the *in vivo* role of platelet Jak2 in atherosclerosis using *ApoE*^*−/−*^ mice with platelet Jak2 deficiency. These mice developed accelerated atherosclerosis in the aortic roots and arches with no significant changes in metabolic parameters. Systemically, there were increased numbers of inflammatory cells including various leukocytes and platelets. Given the prominent role of macrophages in atherogenesis, we also assessed bone marrow–derived macrophages from these mice which exhibited upregulated expression of proinflammatory genes in response to lipopolysaccharide. Furthermore, flow cytometric analysis of the bone marrow showed significant expansion of hematopoietic stem and progenitor cells, suggesting platelet Jak2 effect on hematopoietic stem and progenitor cell expansion. Together, these results show that platelet Jak2 attenuates atherogenesis likely through pleiotropic effects including regulation of inflammation in myeloid cells.

Atherosclerosis is a major underlying pathology in cardiovascular disease (CVD), which is the leading cause of morbidity and mortality globally (1, 2, 3). Atherosclerosis is driven by chronic inflammation involving multiple immune cell types and signaling pathways (1, 4, 5). Atherogenesis is a complex process beginning with low-density lipoprotein (LDL) accumulation in the intima in regions of hemodynamic stress (1). Endothelial cells (ECs) are activated by cytokines and express adhesion molecules such as vascular cell adhesion molecule 1 that increase adherence of monocytes and lymphocytes (6). Monocytes transmigrate into the intima, where they proliferate and differentiate into macrophages that can engulf lipoproteins to form foam cells that eventually die to release lipids and form the necrotic core of the plaque (1). Smooth muscle cells from the media migrate to the intima where they proliferate and secrete extracellular matrix components to form fibrous cap covering the plaque (7). Plaques can become unstable and cause acute thrombosis and rupture, leading to acute ischemia (7).

Platelets are known for their canonical role in hemostasis and thrombosis (8, 9). With endothelial injury, platelets become exposed to thrombogenic factors including collagen and von Willebrand factor and adhere to exposed vessel surface. Platelets degranulate to release various mediators such as ADP, platelet factor 4 (PF4), and TNF-α to further augment their activation, and ADP induces expression of integrin αIIbβ3, mediating platelet adhesion and aggregation (10, 11). Additionally, in the inflammatory milieu of atherogenesis, they are capable of promoting chronic inflammation (12, 13). They interact with ECs to release adhesion molecules and chemokines to further increase activation of ECs and increase immune cell recruitment (14). Platelets also secrete other modulators of inflammation, including various chemokines such as CCL5 (RANTES) to promote the recruitment of monocytes and neutrophils to the atherosclerotic region to promote atherogenesis and progression (15). CXCL4 or (PF4) also causes an increase in the uptake of oxidized LDL by macrophages, contributing to foam cell formation and the lipid core of the plaque (15, 16). Platelets also promote monocyte migration into atherosclerotic plaques and enhance platelet-macrophage aggregate formation in the plaques (17).

The Janus kinase (JAK)/signal transducer and activator of transcription pathway is a major inflammatory signaling pathway that responds to many cytokines (18). Upon ligand binding with its cognate transmembrane receptor, JAK is recruited to the intracellular domains of the receptors and undergo autophosphorylation to become activated (18). Subsequently, signal transducer and activator of transcription proteins are phosphorylated and activated, leading to their dimerization, translocation to the nucleus, and binding to target genes and influencing gene transcription (18, 19). Jak2 is a member of the JAK family of nonreceptor tyrosine kinases that is ubiquitously expressed (20). The effect of Jak2 signaling is highly dependent on the tissue and cell types and the context (18).

*Jak2*^*V617F*^, a well-characterized activating mutation of *Jak2*, is one of the most commonly found mutations in clonal hematopoiesis of indeterminate potential, which has recently been implicated as a major new CVD risk factor. Clonal hematopoiesis of indeterminate potential involves hematopoietic clonal expansion associated with somatic mutations that accumulate with aging (21). Jak2^V617F^ mutation has been associated with over 12-fold increase in incident coronary events (22). The activating mutation in hematopoietic cells has also shown increased susceptibility to atherosclerosis in a hyperlipidemic mouse model (23). Interestingly, the deletion of Jak2 in various cell types also paradoxically leads to increased atherosclerosis. For instance, ApoE-null mice with myeloid-specific Jak2 deletion develop accelerated atherosclerosis (24) despite being protected against high-fat diet–induced obesity, systemic insulin resistance, and inflammation in liver and visceral adipose tissue (25). Furthermore, hepatic Jak2 deletion in male ApoE or LDL receptor-null mice also led to accelerated atherosclerosis (26).

The role of platelet Jak2 in thrombosis has been previously described in the context of atherogenic and vascular injuries (27), as well as hematopoietic and hemostatic function (28, 29, 30). In addition, the role of platelet-Jak2 on mediating thrombopoietin (TPO) signaling to maintain normal circulating platelet levels is well-established (29, 30). However, the role of platelet Jak2 in regulating platelet-mediated inflammatory response involved in atherosclerosis remains unclear. Given that Jak2 is a major inflammatory mediator, we investigated the essential endogenous role of platelet Jak2 in ApoE^−/−^ model of atherosclerosis.

## Results

### ApoE^*−/−*^platelet-Jak2 deficiency leads to accelerated atherosclerosis

To assess the *in vivo* role of *Jak2* in platelets, *ApoE*-null platelet-specific Jak2 KO mice were generated using the Cre-LoxP system under the control of the Pf4 promoter. *ApoE*^*−/−*^ *Pf4cre*^*+*^ *Jak2*^*fl/fl*^ (denoted herein as ApoE^*−/−*^P-Jak2 KO) mice were compared to *ApoE*^*−/−*^*Pf4cre*^*+*^ *Jak2*^*wt/wt*^ littermate controls (denoted herein as ApoE^*−/−*^P-Jak2 WT). Specific deletion of Jak2 in platelets and megakaryocytes in ApoE^*−/−*^P-Jak2 KO mice was shown by flow cytometry and Western blot (Fig. S1).

**Figure 1:**
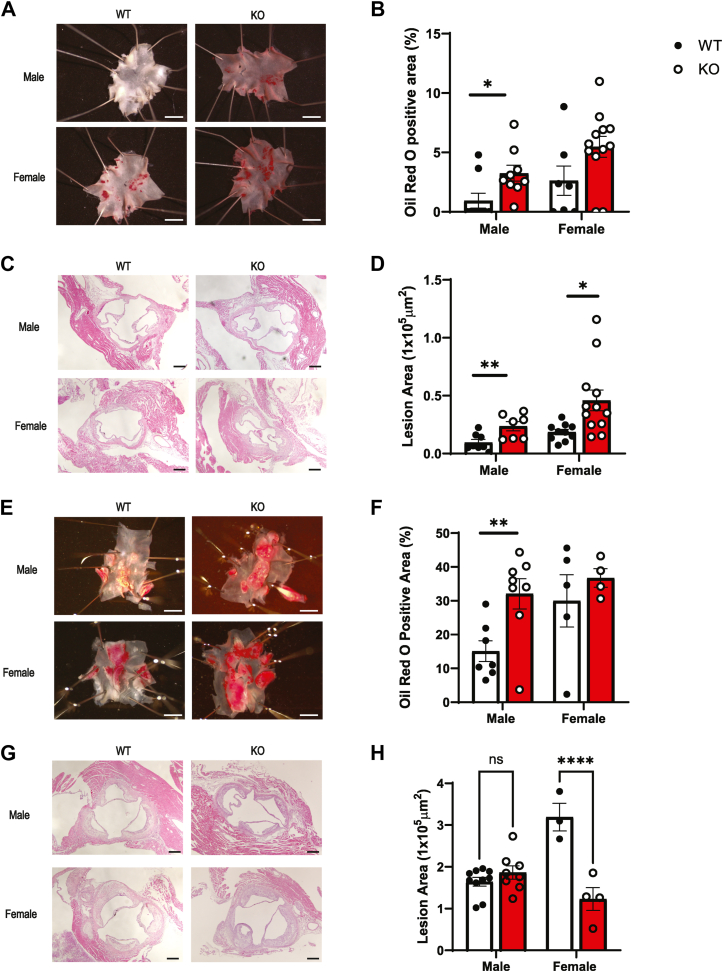
**Accelerated atherosclerosis in ApoE^*−/−*^P-Jak2–deficient mice.***A*, representative photos of en face Oil red O staining of atherosclerotic plaques in the aortic arch after 4 weeks of HCD starting at 6 weeks of age. The scale bar represents 5 mm. *B*, quantification of atherosclerotic area in the aortic arch after 4 weeks of HCD (n = 7–12 per genotype). *C*, representative photos of H&E staining of aortic roots after 4 weeks of HCD. The scale bar represents 200 μm. *D*, quantification of atherosclerotic area in the aortic roots after 4 weeks of HCD (n = 7–12 per genotype). *E*, representative photos of en face Oil red O staining of atherosclerotic plaques in the aortic arches after 16 weeks of HCD for males and 20 weeks of HCD for females. The scale bar represents 5 mm. *F*, quantification of atherosclerotic area in the aortic arch after 16 weeks of HCD for male and 20 weeks of HCD for female (n = 4–8 per genotype). *G*, representative photos of H&E staining of aortic roots after 16 or 20 weeks of HCD. The scale bar represents 200 μm. *H*, quantification of atherosclerotic area in the aortic roots after 16 or 20 weeks of HCD (n = 3–10 per genotype). WT: ApoE^*−/−*^P-Jak2 WT; KO: ApoE^*−/−*^P-Jak2 KO. Statistical analysis: two-tailed unpaired *t* tests were performed. Data are presented as the mean ± SEM, ∗∗*p* < 0.01, ∗*p* < 0.05. HCD, high cholesterol diet; JAK, Janus kinase.

The mice were fed an atherogenic high cholesterol diet (HCD) consisting of 21.2% fat and 0.2% cholesterol beginning at 6 weeks of age. Two time points of HCD were assessed: 4 weeks for early events of atherosclerosis and 16 weeks for male and 20 weeks for female for late events to account for slower plaque development in females. En face of aortic arches stained with Oil red O (ORO) showed significant increase in plaque area in gross appearance (Fig. 1*A*). When quantified, male ApoE^*−/−*^P-Jak2 KO mice exhibited over ∼3-fold increase in atherosclerotic plaque area at 4 weeks of HCD compared to their respective ApoE^*−/−*^P-Jak2 WT littermate controls (Fig. 1*B*). H&E-stained transverse sections of aortic roots also demonstrated significantly increased plaque burden in both male and female ApoE^*−/−*^P-Jak2 KO mice compared to ApoE^*−/−*^P-Jak2 WT controls (Fig. 1, *C* and *D*), with over a 2-fold increase. The increased plaque burden was also observed at the later time point of 16 weeks of HCD for male mice with en face aortic arches showing over 2-fold increase in plaque area in ApoE^*−/−*^P-Jak2 KO mice compared to the WT mice (Fig. 1, *E* and *F*). However, at this time point, there were no significant differences in plaque burden between ApoE^*−/−*^P-Jak2 KO and WT mice in the aortic roots in males, and there was a surprising decrease in the lesion area for female ApoE^*−/−*^P-Jak2 KO mice (Fig. 1, *G* and *H*). In addition, there were no differences in the plaque burden in the descending aortas in both early and late time points in both sexes (Fig. S2). Overall, ApoE^*−/−*^P-Jak2 KO mice displayed increased plaque burden in the aortic arch and root as shown by increased plaque area in both early and late phases of atherosclerosis.

**Figure 2:**
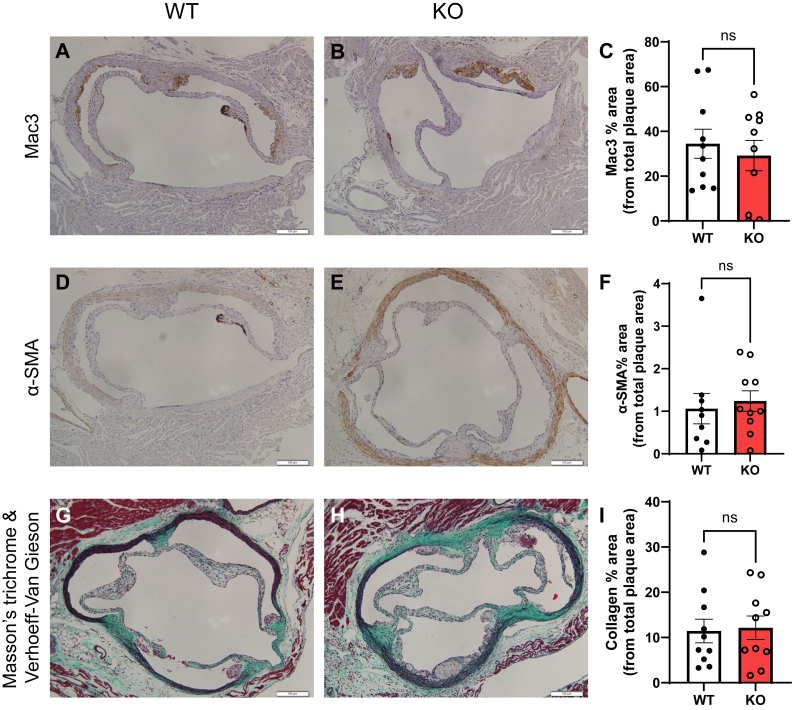
**Plaque characterization of aortic root sections after 4 weeks of HCD.***A* and *B*, representative images of the aortic root sections immunostained with anti-Mac3. The scale bar represents 100 μm. *C*, quantification of Mac3 immunostained area expressed as a percentage of total plaque area (n = 9–10/genotype). *D* and *E*, representative images of aortic root sections immunostained with anti-α-smooth muscle actin (SMA). The scale bar represents 100 μm. *F*, quantification of α-SMA immunostained area expressed as a percentage of total plaque area (n = 9–10/genotype). *G* and *H*, representative images of the aortic root sections combined stained with Masson’s trichrome and Verhoeff-Van Gieson. The scale bar represents 100 μm. *I*, quantification of collagen expressed as a percentage of total plaque area (n = 10/genotype). Statistical analysis: two-tailed unpaired *t* tests were performed. Data are presented as the mean ± SEM. ∗∗∗∗*p* < 0.0001, ∗∗∗*p* < 0.001, ∗∗*p* < 0.01, and ∗*p* < 0.05, ns = not significant. HCD, high cholesterol diet.

Aortic roots, after 4 weeks HCD, were further characterized for macrophage infiltration and plaque progression. There were no differences between ApoE^*−/−*^P-Jak2 KO and WT controls in Mac3-positive area, smooth muscle actin-positive area, and collagen content within plaques (Fig. 2). These findings remained consistent when data were stratified by sex (Fig. S2, *A*–*C*). ApoE^*−/−*^P-Jak2 deficiency does not appear to impact macrophage content in the plaques, vascular smooth muscle cell phenotype and abundance, and indicators of plaque stability and fibrosis. There were also no significant differences between groups in the elastic fiber contents in the arterial walls in the aortic roots (Fig. S2*D*).

### ApoE^*−/−*^platelet-Jak2 KO mice do not exhibit adverse metabolic parameters

**Figure 3:**
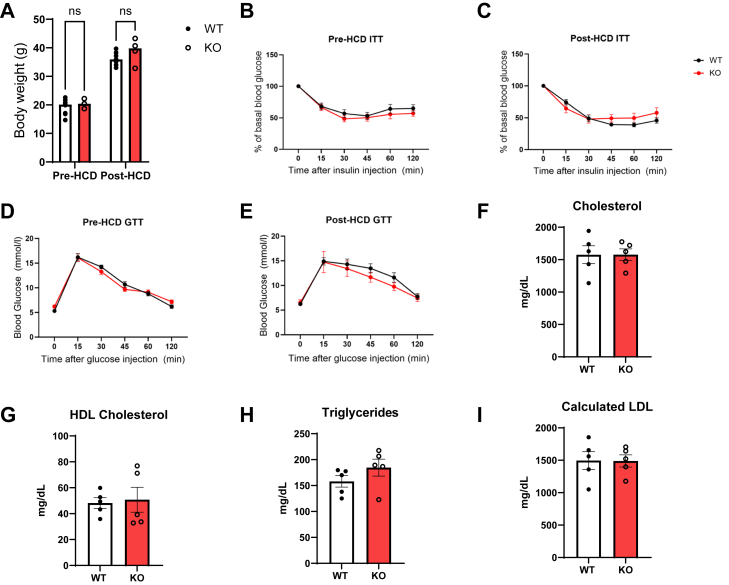
**Metabolic parameters of WT: ApoE**^**−/−**^**P-Jak2 WT and KO: ApoE**^**−/−**^**P-Jak2 KO mice pre- and post-HCD.***A*, pre-HCD and post-HCD body weight of WT and KO male mice after 16 weeks of HCD (n = 3–9 per genotype). *B* and *C*, pre-HCD and post-HCD ITT results in male mice (n = 5–15 per genotype). *D* and *E*, pre-HCD and post-HCD GTT results in male mice. (n = 8–15 per genotype). *F*–*I*, cholesterol, high-density lipoprotein cholesterol, triglycerides, and calculated low-density lipoprotein concentration in male WT and KO mice after 4 weeks of HCD. No significant differences were observed between WT and KO mice for all metabolic parameters. Data are presented as the mean ± SEM. Difference between groups for body weight, ITT, and GTT analyzed for statistical significance by two-way ANOVA with Sidak’s multiple comparisons test. Two-tailed unpaired *t* tests were performed for the lipid panel. Data are presented as the mean ± SEM. ∗∗∗∗*p* < 0.0001, ∗∗∗*p* < 0.001, ∗∗*p* < 0.01, and ∗*p* < 0.05, ns = not significant. GTT, glucose tolerance test; HCD, high cholesterol diet; ITT, insulin tolerance test; JAK, Janus kinase.

Given that metabolic abnormalities such as diabetes and obesity are known risk factors for CVD, we assessed metabolic parameters which may have contributed to the increased atherosclerosis in ApoE^*−/−*^P-Jak2 KO mice. There was no difference in weight gain in ApoE^*−/−*^P-Jak2 KO mice compared to the ApoE^*−/−*^P-Jak2 WT controls in male mice after 16 weeks of HCD (Fig. 3*A*). Moreover, there were no significant differences in glucose homeostasis either pre- or post-HCD as assessed by glucose and insulin tolerance tests in both male and female mice (Fig. 3, *B*–*E* and S3). Plasma lipid analysis showed no significant differences in the levels of total serum cholesterol, high-density lipoprotein cholesterol, triglycerides, and calculated LDL between the ApoE^*−/−*^P-Jak2 KO mice and the ApoE^*−/−*^P-Jak2 WT controls (Fig. 3, *F*–*I*). Together, these results show that metabolic abnormalities did not likely contribute to the increased atherosclerotic plaque burden in ApoE^*−/−*^P-Jak2 KO mice.

### ApoE^−/−^platelet-Jak2 KO mice have increased number of platelets

**Figure 4:**
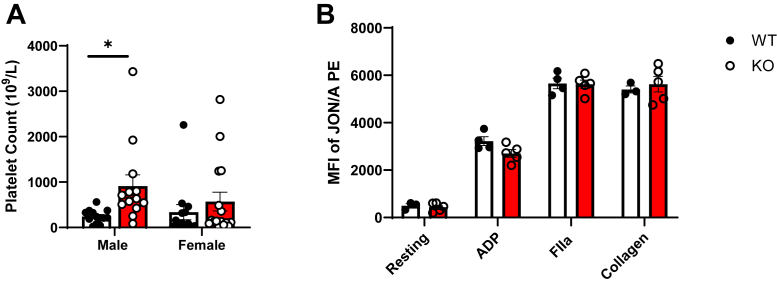
**Platelet counts and activation in WT: ApoE^*−/−*^P-Jak2 WT and KO: ApoE^*−/−*^P-Jak2 KO mice following HCD.***A*, platelet count of WT and KO mice after 4 weeks of HCD starting at 6 weeks of age. Platelet numbers were significantly elevated in male KO mice compared to male WT mice, while no differences were observed in female mice. (n = 13–16 per genotype). *B*, JON/A-PE binding to resting platelets and platelets activated with ADP, FIIa, and collagen in WT and KO mice after 12, 17, and 22 weeks of HCD using flow cytometry (n = 4–5 per genotype). No significant differences in integrin αIIbβ3 activation were observed between WT and KO mice across stimulation with different agonists. Statistical analysis: two-tailed unpaired *t* tests were performed. Data are presented as the mean ± SEM. ∗∗∗∗*p* < 0.0001, ∗∗∗*p* < 0.001, ∗∗*p* < 0.01, and ∗*p* < 0.05 MFI, mean fluorescence intensity; HCD, high cholesterol diet; JAK, Janus kinase.

We next assessed the effects of platelet Jak2 on their cell count in circulation. After 4 weeks of HCD, male ApoE^*−/−*^P-Jak2 KO mice exhibited significantly increased levels of platelets compared to WT controls. (Fig. 4*A*). This is consistent with previous findings that Jak2 deficiency in platelets lead to an increase in platelet production due to dysregulation in Tpo turnover leading to expansion of hematopoietic stem cells (HSCs) and megakaryocyte-biased progenitors (27). We then investigated the role of Jak2 in platelet activation by measuring expression of their activation marker, integrin αIIbβ3, at rest and following stimulation with ADP, thrombin (FIIa), or collagen. Platelet integrin αIIbβ3 turns to a high affinity conformation state with activation, mediating platelet adhesion and aggregation (31). No significant difference in platelet αIIbβ3 expression was observed between ApoE^*−/−*^P-Jak2 KO and WT mice as assessed by flow cytometry in either resting or ADP, FIIa, or collagen-activated platelet-rich plasma (PRP) (Fig. 4*B*). This indicates that platelet Jak2, while important in determining platelet counts, is not essential for regulating the threshold for stimulation in response to various platelet-activating factors.

### Increased number of peripheral leukocytes and altered bone marrow composition in ApoE^*−/−*^platelet-Jak2 KO mice

**Figure 5:**
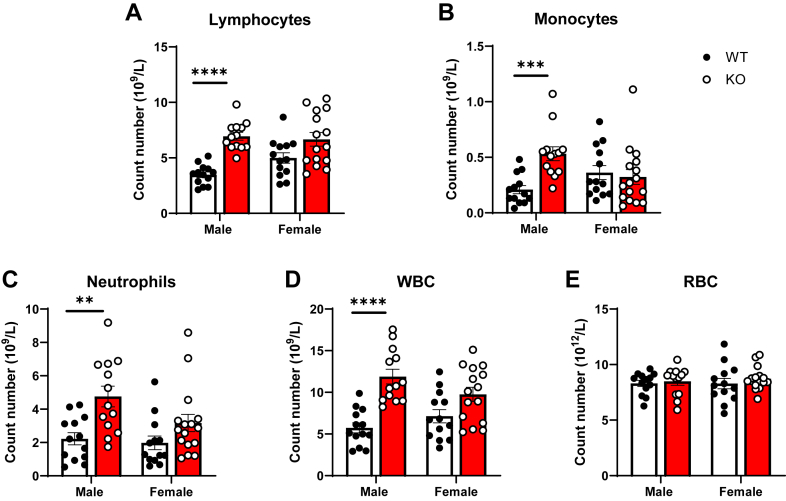
**Blood cell counts of WT: ApoE**^***−/−***^**P-Jak2 WT and KO: ApoE**^***−/−***^**P-Jak2 KO mice after 4 weeks of HCD starting at 6 weeks of age.***A–E,* The number of lymphocytes, monocytes, neutrophils, and total white blood cells (WBCs) were significantly elevated in male KO mice, with no differences in female KO mice compared to their respective WT controls. No differences between genotypes were present in the number of red blood cells (RBCs) in both male and female mice. Statistical analysis: two-tailed unpaired t tests were performed. (n = 13–16 per genotype). Data are presented as the mean ± SEM, ∗∗∗∗*p* < 0.0001, ∗∗∗*p* < 0.001, ∗∗*p* < 0.01, and ∗*p* < 0.05. HCD, high cholesterol diet; JAK, Janus kinase.

To investigate the impact of platelet Jak2 on immune populations, we examined the peripheral blood of ApoE^*−/−*^P-Jak2 KO mice. Male ApoE^*−/−*^P-Jak2 KO mice exhibited significant increases in the cell number of lymphocytes, monocytes, and neutrophils (Fig. 5, *A*–*D*), whereas no alterations in hematopoiesis were observed in female ApoE^*−/−*^P-Jak2 KO mice. Notably, erythrocyte levels remained unchanged in both sexes (Fig. 5*E*).

**Figure 6:**
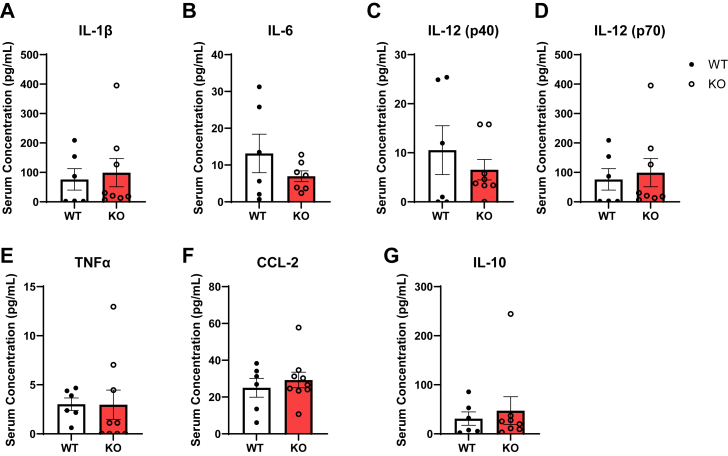
**Serum concentrations of cytokines from WT: ApoE^*−/−*^P-Jak2 WT and KO: ApoE^*−/−*^P-Jak2 KO mice after 4 weeks of HCD starting at 6 weeks of age (n = 5–10 per genotype).** There were no significant differences in the serum concentrations of circulating (*A*) IL-1β, (*B*) IL-6, (*C*) IL-12 (p40), (*D*) IL-12 (p70), (*E*) TNFα, (*F*) CCL-2, and (*G*) IL-10 between WT and KO mice. Statistical analysis: two-tailed unpaired *t* tests were performed. Data are presented as the mean ± SEM. HCD, high cholesterol diet; JAK, Janus kinase.

The serum levels of major proinflammatory and anti-inflammatory cytokines were also measured to assess whether platelet Jak2 deletion impacts the levels of circulating inflammatory mediators. Multiplex analysis revealed no significant differences in circulating proinflammatory cytokine levels including IL-1β, IL-6, TNFα, CCl2, and IL-12 or anti-inflammatory cytokine IL-10 between ApoE^*−/−*^P-Jak2 KO and WT mice after 4 weeks of HCD (Fig. 6). This suggests that platelet Jak2 does not significantly impact systemic cytokine concentrations in atherogenic conditions.

**Figure 7:**
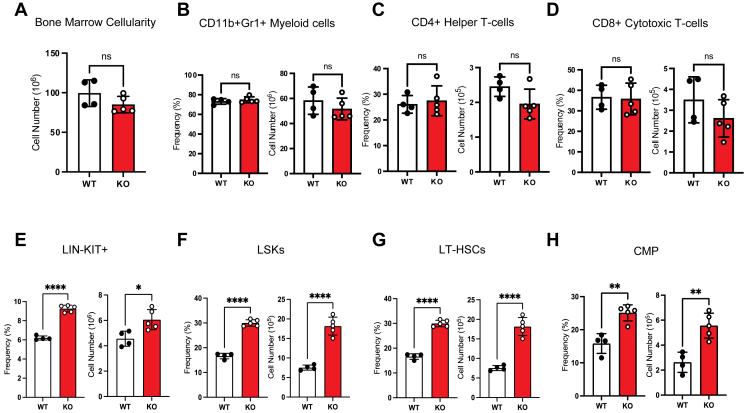
**Frequency and cell number of hematopoietic progenitor and lineage committed cells in the BM of ApoE^*−/−*^P-Jak2–deficient mice.***A*, BM cellularity of male mice 4 weeks of age (n = 4–5 per genotype). *B*-*D*, lineage-committed cells and monocytes of WT: ApoE^*−/−*^P-Jak2 WT and KO: ApoE^*−/−*^ P-Jak2 KO mice. *E*-*H*, progenitor cells in BM of male mice 4 weeks of age (n = 4–5 per genotype). The absolute numbers and frequency of LIN^-^KIT^+^, LSKs, LT-HSCs, and CMPs were significantly increased. Statistical analysis: two-tailed unpaired t tests were performed. Data are presented as the mean ± SEM, ∗∗∗∗*p* < 0.0001, ∗∗∗*p* < 0.001, ∗∗*p* < 0.01, and ∗*p* < 0.05. BM, bone marrow; CMP, common myeloid progenitor; HCD, high cholesterol diet; JAK, Janus kinase; LIN^-^KIT^+^, lineage negative cKit^+^; LT-HSC, long-term hematopoietic stem cell; LT-HSC, long-term HSC, hematopoietic stem cell.

To explore whether megakaryocyte and/or platelet Jak2 might influence the bone marrow (BM) microenvironment governing circulating immune cell populations, we further evaluated hematopoiesis in the BM. The hematopoietic progenitor compartment in the BM at 4 weeks of age showed a comparative total cellularity between ApoE^*−/−*^P-Jak2 KO and control littermates (Fig. 7*A*), as well as similar mature leukocyte populations across all lineages (Fig. 7, *B*–*D*). Intriguingly, we identified a significant expansion in hematopoietic stem and progenitor cells (HSPCs) in ApoE^*−/−*^P-Jak2 KO mice. Specifically, we noted significant increases in lineage negative cKit^+^, lineage negative Sca-1^+^ cKit^+^ hematopoietic progenitor, and long-term HSCs (Fig. 7, *E*–*G*). Furthermore, oligopotent common myeloid progenitors were also increased in ApoE^*−/−*^P-Jak2 KO mice compared to ApoE^*−/−*^P-Jak2 WT controls (Fig. 7*H*). Thus, striking increases in peripheral leukocyte populations are likely driven by HSPC expansion in ApoE^*−/−*^P-Jak2 KO mice.

### ApoE^−/−^platelet-Jak2 KO mice show increased activation of BM-derived macrophages

**Figure 8:**
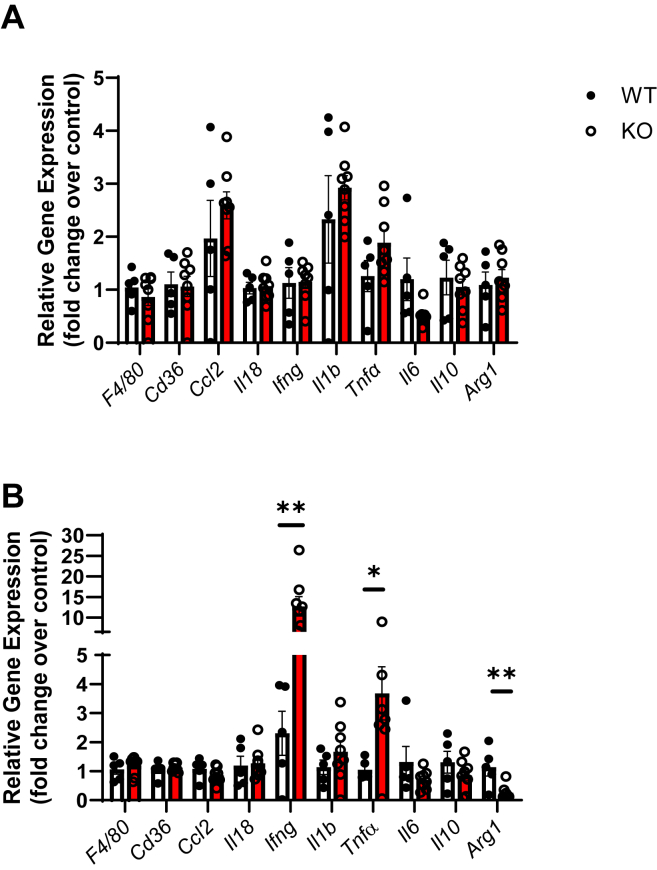
**RT-qPCR results of BMDMs from WT: ApoE**^**−/−**^**P-Jak2 WT and KO: ApoE**^**−/−**^**P-Jak2 KO mice after 6 weeks of HCD.***A*, quantitative RT-PCR results of basal/unstimulated BMDMs from female mice after 6 weeks of HCD. Relative gene expression is normalized to WT BMDMs. There were no significant differences in the relative gene expression of macrophage markers (*F4/80* and *Cd36*), inflammatory markers (*Ccl2, Il18, Ifng, Tnfα*), or anti-inflammatory markers (*Il10, Arg1*) between the BMDMs of KO and WT mice. (n = 5–8 per genotype). *B*, quantitative RT-PCR results of BMDMs stimulated with LPS from female mice after 6 weeks of HCD. Relative gene expression is normalized to WT BMDMs. Significant increase in proinflammatory gene expression (*Ifng* and *Tnfα*) and significant decrease in anti-inflammatory gene expression (*Arg1*) were observed. (n = 5–8 per genotype). Statistical analysis: two-tailed unpaired *t* tests were performed. Data are presented as the mean ± SEM, ∗∗*p* < 0.01, ∗*p* < 0.05. BMDM, bone marrow–derived macrophage; HCD, high cholesterol diet; JAK, Janus kinase; LPS, lipopolysaccharide.

To further investigate the effects of platelet/megakaryocyte Jak2 in the fate and function of macrophage/myeloid cells and the associated peripheral inflammatory state, we examined the BM-derived macrophages (BMDM) at baseline and in response to lipopolysaccharide (LPS). No difference in the relative expression of gene markers of macrophages, or of proinflammatory or anti-inflammatory states were observed between BMDMs of ApoE^*−/−*^P-Jak2 KO and ApoE^*−/−*^P-Jak2 WT mice under unstimulated conditions (Fig. 8*A*). Intriguingly, with LPS simulation, significant increases in expression of proinflammatory genes, *Ifng* and *Tnfa*, and decreases in anti-inflammatory gene, *Arg1*, were observed (Fig. 8*B*). These data suggest that ApoE^*−/−*^P-Jak2 deficiency upregulated the response of BMDM to a proinflammatory stimulus of macrophage/myeloid cells. Together, our data show direct and indirect effects of platelet Jak2 in mediating systemic inflammatory response that likely led to accelerated atherosclerosis in ApoE^*−/−*^P-Jak2 KO mice.

## Discussion

In this study, we investigated the *in vivo* role of platelet Jak2 in atherosclerosis. ApoE-null mice with platelet-specific Jak2 deletion were used to show that Jak2 in platelets plays a critical role in regulating atherosclerosis. Mice with ApoE^*−/−*^P-Jak2 deficiency developed accelerated atherosclerosis in both sexes, while not displaying significant metabolic abnormalities or differences in plaque characteristics. Increased platelet and leukocyte counts, but not platelet activation or function were present in mice with ApoE^*−/−*^P-Jak2 deletion following stimulation with various platelet-activating factors. This was accompanied by changes in the HSPCs in the BM that are associated with increased chronic inflammation. These findings suggest that increased atherosclerosis burden may be attributed by the increased inflammation and hematopoiesis in the BM. In addition, platelet Jak2 plays a significant role in determining the inflammatory response of macrophages as shown by BMDM from ApoE^*−/−*^P-Jak2 KO mice.

Jak2 is a critical downstream mediator of inflammatory and metabolic signaling that functions in a highly cell type– and context-specific manner (18, 25, 32, 33, 34). We have previously shown that deficiency of Jak2 in adipocytes led to exacerbated systemic insulin resistance and increased adiposity due to impaired lipid homeostasis (33); and hepatocyte-specific Jak2 KO mice exhibit fatty liver and exacerbated atherosclerosis (26). On the other hand, macrophage-specific Jak2 KO mice are protected against high-fat diet–induced systemic insulin resistance, obesity, and inflammation (25), but paradoxically also show increased atherosclerosis in an ApoE-null model (24). The specific function of Jak2 in different tissues and cell types warranted the investigation of platelet Jak2 on inflammation and atherosclerosis. Despite Jak2 being a known mediator of inflammatory signaling, our work shows that deficiency of platelet Jak2 leads to accelerated atherosclerosis with upregulation of inflammation.

Platelets, in addition to their role in hemostasis and atherothrombosis, can drive atherogenesis through other mechanisms (35). Release of chemokines by activated platelets can stimulate and recruit leukocytes that subsequently also express inflammatory mediators (36, 37, 38) and increase the recruitment of leukocytes to the activated endothelium during atherogenesis (39). Activated platelets also upregulate expression of their adhesion receptors (40). Our flow cytometric results show that there are no significant changes in platelet integrin αIIbβ3 expression levels with Jak2 deficiency, following ADP stimulation, which is consistent with the earlier report (28). Platelet integrin αIIbβ3 is a fibrinogen receptor that is essential for platelet aggregation, and interaction of activated platelets to receptors on the endothelium can thereby promote atherosclerosis (37, 40). The lack of difference in platelet stimulation by ADP, FIIa, and collagen suggests that the increase in atherosclerosis observed in ApoE^*−/−*^P-Jak2 KO mice are likely attributable to other factors than the mechanism of integrin αIIbβ3 activation. Activation studies of other platelet receptors such as P-selectin and phosphatidylserine (41), and measurement of platelet thromboxane B2 may further elucidate the state of platelet activation in ApoE^*−/−*^P-Jak2 KO mice and its contribution to the increase in atherosclerosis. Notably, although platelets have been generally considered as proinflammatory cells, emerging evidence shows their expanding regulatory roles to also promote immunosuppression, further demonstrating the growing multifaceted roles that platelets play in the regulation of atherogenesis (42, 43). Thus, elucidation of other signaling pathways in platelets may further clarify the proinflammatory state and accelerated atherosclerosis that we report in ApoE^*−/−*^P-Jak2 KO mice.

Jak2^V617F^ activating mutation, which causes constitutively increased Jak2 signaling, in LDL receptor-null mice led to increased atherosclerosis in the aortic root compared to WT controls (23). This was due to increased tendency of erythrophagocytosis and defective efferocytosis, leading to unstable plaque. Indeed, clonal hematopoiesis that occurs with Jak2^V617F^ has been identified as a major CVD risk factor, being associated with a 12-fold increase in coronary events (22). Interestingly, our results indicate that deletion of Jak2 in platelets also leads to increased atherosclerosis risk with increased plaque burden in the aorta. This may be attributable to the enhanced platelet activation from increase in number. Platelet Jak2 is an important player in the Tpo/Mpl signaling axis that determines platelet production. When Tpo that is produced in the liver binds to HSCs and megakaryocytic progenitors, it is internalized and degraded (29, 44). However, with Jak2 deletion, degradation may be compromised, leading to increased availability of Tpo in the BM and subsequent expansion of megakaryocyte progenitors, resulting in thrombocytosis (28). Thus, excessive platelet number from Jak2 deficiency may lead to increased atherosclerotic risk as shown by increased CVD and acute coronary syndromes (35, 45).

In addition to the impact of Jak2 deficiency on platelet number, monocytes are affected by the lack of Jak2 in platelets. BMDM from ApoE^*−/−*^P-Jak2 KO mice expressed significantly increased levels of proinflammatory genes and decrease in anti-inflammatory gene in response to LPS stimulation. This suggests that megakaryocytes can affect monocytes in the BM to alter their inflammatory response. This upregulation may lead to increased susceptibility of macrophage activation during atherogenesis, including foam cell formation, cytokine and chemokine release, and necrotic core formation (46). In addition, ApoE^*−/−*^P-Jak2 KO mice show a significant increase in leukocytes including monocytes after 4 weeks of HCD, which is also an indicator of a proinflammatory state and predictor of CVD (38, 39, 40, 47). Interestingly, circulating cytokine measurements showed no significant differences between ApoE^*−/−*^P-Jak2 KO and WT mice. Similar levels of proinflammatory (IL-1β, IL-6, C, IL-12 (p40), IL-12 (p70), TNFα, CCL-2) and anti-inflammatory (IL-10) cytokines were present between the ApoE^*−/−*^P-Jak2 KO mice and their WT controls after 4 weeks of HCD. This suggests that platelet Jak2 deficiency does not significantly alter systemic inflammation at least as measured through the modulation of these circulating cytokines. Thus, the role of P-Jak2 in the inflammatory response in atherosclerosis may be confined to the vasculature, the atherosclerotic lesion, or the BM.

Our results show that in the BM of ApoE^*−/−*^P-Jak2 KO mice, there was an increase in number and frequency of lineage negative Sca-1^+^ cKit^+^ hematopoietic progenitor cells, LT-HSCs, and common myeloid progenitors. HSPCs in the BM can sense and respond to chronic inflammation through increased proliferation and myeloid skewing (45, 46). In line with the HSPC expansion seen in the ApoE^*−/−*^P-Jak2 KO mice, HSPCs from humans with atherosclerotic CVD have been shown to have enhanced proliferative potential (44). Our flow cytometry results indicate that platelet/megakaryocyte Jak2 deficiency affects hematopoiesis at the level of the BM that increases the inflammatory state of the HSCs, which can subsequently lead to increased number of peripheral inflammatory cells to ultimately promote the development of atherosclerosis.

Our study also explored the sex-specific effects of P-Jak2 in atherosclerosis through analyzing the male and female mice separately. The male mice displayed more significantly increased plaque burden in the aortic arches and roots compared to the females. Interestingly, platelet, lymphocyte, monocyte, and neutrophil counts were significantly elevated in male ApoE^*−/−*^P-Jak2 KO mice, whereas there were no differences in the females. These striking differences suggest a greater impact of P-Jak2 deficiency in males in the upregulation of inflammation leading to increased peripheral blood cells and increased atherosclerosis burden. Estrogen has been shown to be atheroprotective in humans and mice through effects including vasodilation and decreased inflammatory response (48, 49). Paradoxically, studies report female ApoE-null mice are affected by greater atherosclerotic plaque burden (50, 51). Thus, further elucidation of the mechanism by which P-Jak2 intersects with sex hormone signaling or other factors may reveal valuable information about the sex-dependent effects in the pathogenesis of atherosclerosis.

In conclusion, platelet Jak2 deficiency caused accelerated atherosclerosis through increased inflammatory response of platelets, macrophages, and HSPCs. Both the number of platelets and proinflammatory response of macrophages were increased with ApoE^*−/−*^P-Jak2 deficiency. Changes in the BM were evident with significantly increased HSPC number and frequency in the KO mice. Further exploration into the molecular mechanism of increase in inflammation may elucidate the role of ApoE^−/−^P-Jak2 in atherosclerosis.

## Experimental procedures

### Generation of ApoE^−/−^platelet-specific Jak2-KO mice

Platelet-specific Jak2 KO mice were generated using the Cre-LoxP system. Platelet and megakaryocyte-specific Cre recombinase-expressing (PF4-Cre^+^) mice (Jackson Laboratory: 008535) were crossed with Jak2 floxed mice (Dr Kay-Uwe Wagner) with loxP sites flanking the first coding exon of the Jak2 gene (52). The resulting *PF4 Cre*^*+*^ *Jak2*^*wt/fl*^ mice were then interbred to generate *PF4 Cre*^*+*^ *Jak2*^*fl/fl*^ mice that were crossed with *ApoE*^*−/−*^ mice (JAX: B6.129P2-Apoetm1Unc/J). Finally, *PF4 Cre*^*+*^ *ApoE*^*−/−*^ *Jak2*^*wt/fl*^ were interbred to generate *PF4-Cre*^*+*^ *Jak2*^*fl/fl*^*ApoE*^*−/−*^ (ApoE^−/−^P-Jak2 KO) and *PF4-Cre*^*+*^ *Jak2*^*wt/wt*^*ApoE*^*−/−*^ (ApoE^−/−^P-Jak2 WT).

The mice were maintained in a pathogen-free and temperature-controlled animal facility at the Princess Margaret Cancer Research Tower (University Health Network) with 12-h light and dark cycle and free access to food and water. Both female and male mice were fed normal chow diet until 6 weeks of age and then switched to an atherogenic HCD containing 21.2% fat and 0.2% cholesterol (TD.88137 Teklad Custom Diets). Early events of atherosclerosis were examined after 4 weeks of HCD and late events were examined after 16 weeks of HCD for male and 20 weeks of HCD for female mice. Two different durations of HCD for different sexes were to ensure adequate plaque formation in females as it was noticed that the female mice in our animal colony generally developed atherosclerosis at a slower rate. All animal experimental protocols were approved and performed in accordance with the guidelines of the Canadian Council on Animal Care and regulations established by the Toronto General Hospital Research Institute Animal Care Committee (AUP 2862).

### Metabolic analyses

Intraperitoneal glucose and insulin tolerance tests were performed as previously described (53). For glucose tolerance test, the mice were fasted overnight, from 5:00 PM to 9:00 AM, and given a glucose dose of 1.0 g/kg bodyweight intraperitoneally. For insulin tolerance test, mice were fasted for 4 h before intraperitoneal injection of insulin at a dose of 0.75 units/kg body weight. All blood glucose levels were determined from tail venous blood using an automated glucometer. The weights of mice on HCD were measured biweekly.

### Western blot

Western blots on platelets and liver lysates were performed as previously described (24). Mouse platelets (5 × 10^7^) and liver lysates (60 ug) were separated by SDS-PAGE and then transferred to a polyvinylidene fluoride membrane. The membranes were blocked with 5% skim milk and probed with an anti-JAK2 (CST#3230; 1:1000) (1:500 dilution) and GAPDH (CST2118; 1:1000), followed by a horseradish peroxidase-conjugated anti-rabbit secondary antibody (CST#7074; 1:5000–10000) and imaged using an ECL chemiluminescent reagent. Protein expression levels were quantified using ImageJ software (https://imagej.net/ij/). To determine relative signal intensity, peak areas corresponding to each band were measured. All protein levels were normalized to the intensity of corresponding loading controls.

### Atherosclerotic plaque assessment and quantification

Atherosclerotic plaque burden was determined as previously described (24). Atherosclerotic plaques were examined in the aortic roots and arches at 4 and 16 weeks or 20 weeks of HCD to assess early and late stages of plaque development, respectively. At their endpoints, mice were perfused with PBS then 4% paraformaldehyde prior to extraction of the heart and the aorta. En face sections of aortic arches were stained with ORO to visualize lipid-rich plaques. Adipose tissue attached to the outside of the aorta was removed prior to ORO staining. Aortas were stained with ORO stock solution, comprising of 0.3 g/10 ml isopropanol with 3:2 ratio of stock ORO with water, for 30 min then washed 2 times with 60% isopropanol. Plaque area of aortic arches and descending aortas were quantified as percentage of ORO-positive area over total surface area using ImageJ software.

Cross-sections of aortic roots and sagittal sections of aortic arches were stained with H&E to also visualize atherosclerotic plaque. Quantification of plaque area in μm^2^ was measured using ImageJ software. Plaque area was defined as the area extending from the internal elastic lamina to the luminal edge of the plaque.

### Immunohistochemistry

Aortic roots were fixed in 4% paraformaldehyde in PBS. Tissue sections were stained with anti-Mac3 antibody, anti-α-SMA antibody, and Masson’s trichrome and Verhoeff Van Gieson stain in the STTARR facility as previously described (24, 54). Quantification was performed using ImageJ software, applying same color thresholds across all images for each stain.

### Platelet activation assay

Blood was collected by retro-orbital bleeding of mice into 3.8% sodium citrate tubes (BD Vacutainer). To isolate platelets, whole blood was centrifuged at 300*g* for 7 min to obtain PRP. Platelet concentration in the PRP was measured using a Z2 Series Coulter Counter (Beckman Coulter) and diluted to 1 × 10^7^ cells/ml in Tyrode’s buffer (129 mM sodium chloride, 2.9 mM potassium chloride, 0.34 mM disodium phosphate, 12 mM sodium bicarbonate, 20 mM Hepes, 5 mM glucose, and 1 mM magnesium chloride; pH 7.4) supplemented with 2.5 mM calcium chloride.

Next, 50 μl of the platelet suspension (1 × 10^7^ cells/ml with Ca^2+^) was aliquoted into individual 5 ml polystyrene flow cytometry tubes. Platelet activation was induced by stimulating platelets at room temperature for 2 to 10 min with one of three agonists: 10 μM adenosine 5′-diphosphate sodium salt (Sigma-Aldrich), 0.05 U/ml thrombin from bovine plasma (Sigma-Aldrich), or 2 μg/ml CHRONO-PAR collagen (Chrono-log Corporation).

Following stimulation, platelets were stained with phycoerythrin-conjugated anti-mouse CD41/CD61 antibody (1:10 dilution, clone JON/A, Chemfree) for 15 min in the dark at room temperature. Samples were then diluted with 500 μl of Tyrode’s buffer lacking calcium and analyzed using a Sony SP6800 spectral cytometer. Platelet activation was quantified as the median fluorescence intensity of JON/A binding over 5000 events. Gel-filtered platelets were diluted to 1 × 10^7^ cells/ml, supplemented with, and then allowed to rest for 30 min at room temperature.

### BM flow cytometry

The hip, femur, and tibia bones of 4-week-old mice were placed in a culture plate with fluorescence-activated cell sorting (FACS) buffer (1× PBS and 2% fetal bovine serum) on ice. After removal of flesh and muscle, bones were crushed using mortar and pestle, and resulting cell suspension was filtered through 70 um mesh into a 50 ml falcon tube. Cell suspension was centrifuged at 1500 rpm for 5 min and the cell pellet was resuspended with 3 ml lysis buffer to lyse red blood cells and incubated at room temperature for 3 min. Twenty milliliters of FACS buffer was added and cell suspension was centrifuged again at 1500 rpm for 5 min and the pellet was resuspended in 10 ml FACS buffer and put through another 70-um filter. Cells were incubated with antibodies run through the flow cytometer BD LSR. The acquired data were analyzed using FlowJo (https://www.flowjo.com/).

### BMDM isolation and culture

BM was collected from the femur and tibia after 4 weeks of HCD and cultured in 40 ng/ml of M-CSF (PeproTech) in RPMI 1640 media (Gibco) supplemented with 10% fetal bovine serum (Sigma-Alrich), 1% penicillin-streptomycin (Gibco), and 1% L-glutamine (Wisent) for the first 5 days. Next, the cells were cultured in Dulbecco's modified Eagle's medium (Wisent) overnight before being harvested. BMDM was subsequently stimulated with 10 ng/ml of LPS (InvivoGen) for 6 h prior to RNA isolation.

### Quantitative RT-PCR

Total RNA was isolated from BMDM using an RNeasy mini kit (Qiagen). Four hundred nanograms of RNA was then reverse-transcribed with random primers using the M-MLV reverse transcriptase enzyme (Invitrogen). Quantitative polymerase chain reaction was performed using SYBR green master mix (Applied Biosystems) under standard conditions using 7900HT Fast-Real-Time PCR machine. Each sample was run in a 10 μl reaction volume and was performed in triplicates. The housekeeping gene 18S was used to normalize the relative expression level of each gene. Primer sequences are listed in Table 1.

**Table 1:** Primer Sequences for qPCR

Gene	Forward sequence	Reverse sequence
F4/80	CGTGTTGTTGGTGGCACTGTGA	CCACATCAGTGTTCCAGGAGAC
Cd36	GGACATTGAGATTCTTTTCCTCTG	GCAAAGGCATTGGCTGGAAGAAC
Ccl2	GCTACAAGAGGATCACCAGCAG	GTCTGGACCCATTCCTTCTTGG
Il18	GACAGCCTGTGTTCGAGGATATG	TGTTCTTACAGGAGAGGGTAGAC
Ifnγ	CAGCAACAGCAAGGCGAAAAAGG	TTTCCGCTTCCTGAGGCTGGAT
Il1β	TGGACCTTCCAGGATGAGGACA	GTTCATCTCGGAGCCTGTAGTG
Tnf*α*	GGTGCCTATGTCTCAGCCTCTT	GCCATAGAACTGATGAGAGGGAG
Il6	TACCACTTCACAAGTCGGAGGC	CTGCAAGTGCATCATCGTTGTTC
Il10	CGGGAAGACAATAACTGCACCC	CGGTTAGCAGTATGTTGTCCAGC
Arg1	CATTGGCTTGCGAGACGTAGAC	GCTGAAGGTCTCTTCCATCACC
18s	AGTCCCTGCCCTTTGTACACA	CGATCCGAGGGCCTCACTA

qPCR, quantitative PCR.

### Blood and serum analyses

Blood was collected from mice *via* cardiac puncture into EDTA-lined tubes and placed on ice. Concentrations of white blood cells, lymphocytes, monocytes, neutrophils, red blood cells, and platelets were measured using a hematology analyzer (VetScan HM5 v2.31) by the Animal Resource Centre at University Health Network. Serum IL-1β, IL-6, IL-12 (p70), CCL-2, TNFα, IL-10, and IL-12 (p40) were measured using Milliplex Mouse Cytokine/Chemokine Magnetic Bead Panel (Millipore). Serum total cholesterol, triglycerides, and high-density lipoprotein cholesterol, and calculated LDL measurements were performed at The Centre for Phenogenomics.

## Data availability

Datasets used and analyzed in the study are available from the corresponding author on request.

## Supporting information

This article contains supporting information.

## Conflict of interest

The authors declare that they have no conflicts of interest with the contents of this article.
