# Supplementary material for: Platelet Jak2 deficiency accelerates atherosclerosis with increased inflammatory response
Source: J Biol Chem. 2025 Aug 16;301(10):110603. doi: 10.1016/j.jbc.2025.110603 (PMC12624798; doi:10.1016/j.jbc.2025.110603)
Supplement: Supplementary Figures [file mmc2.pdf]

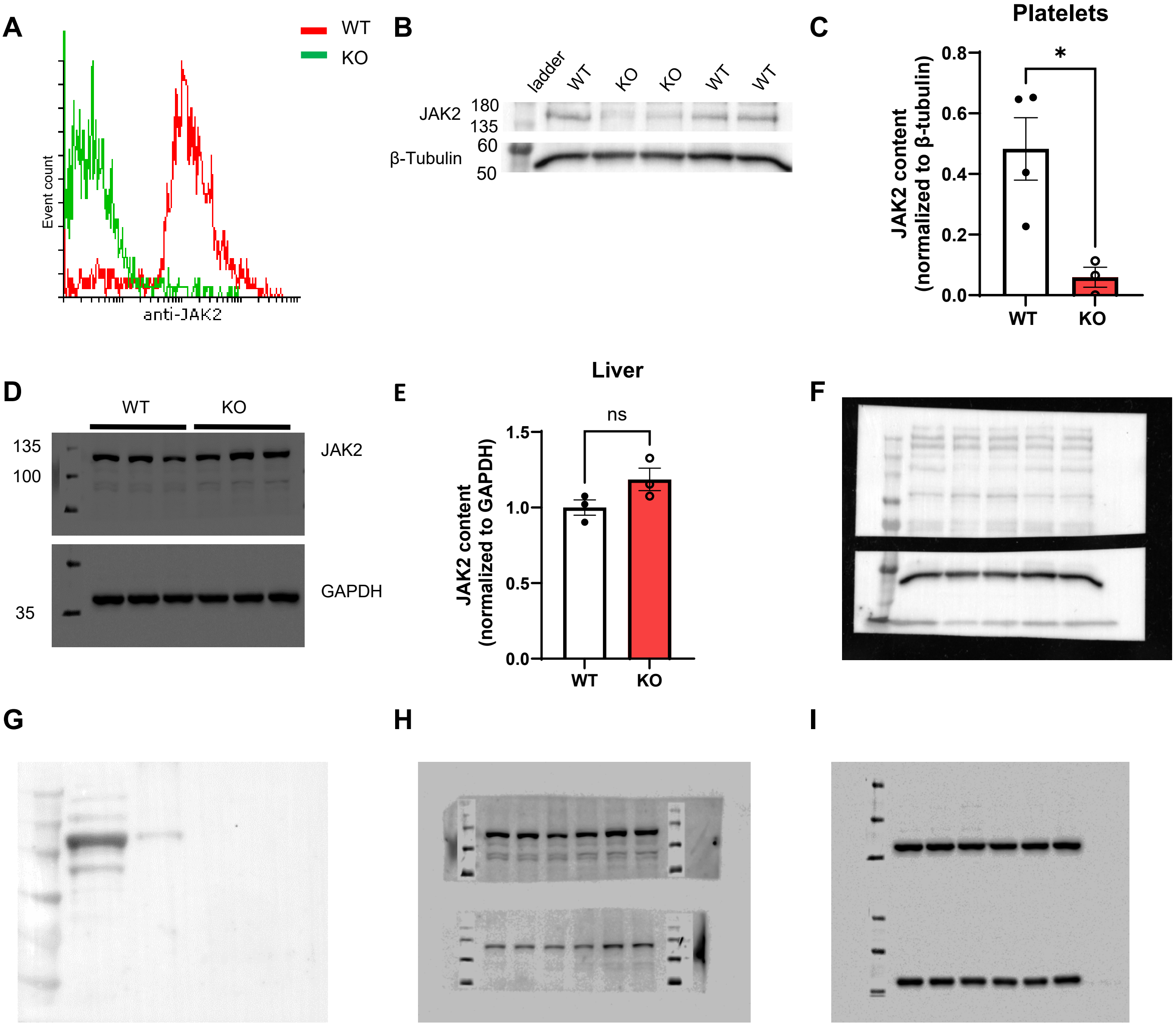

Figure S1. Jak2 expression in WT: ApoE$^{-/-}$P-Jak2 WT and KO: ApoE$^{-/-}$P-Jak2 KO mice. A) Anti-Jak2 antibody binding in permeabilized platelets using flow cytometry. B) Western blot images of Jak2 content in platelets with β-tubulin loading controls. C) Quantification of Jak2 content in platelets normalized to β-tubulin controls. D) Jak2 expression in liver lysates with GAPDH loading controls. E) Quantification of Jak2 content in liver normalized to GADPH controls. F,G) Uncropped western blots of Jak2 and β-tubulin content in platelets. H) Uncropped western blot of Jak2 expression in liver lysates. I) Uncropped western blot of GADPH control in liver lysates. Statistical analysis: two-tailed unpaired t tests were performed. Data are presented as the mean ± SEM, *P<0.05.

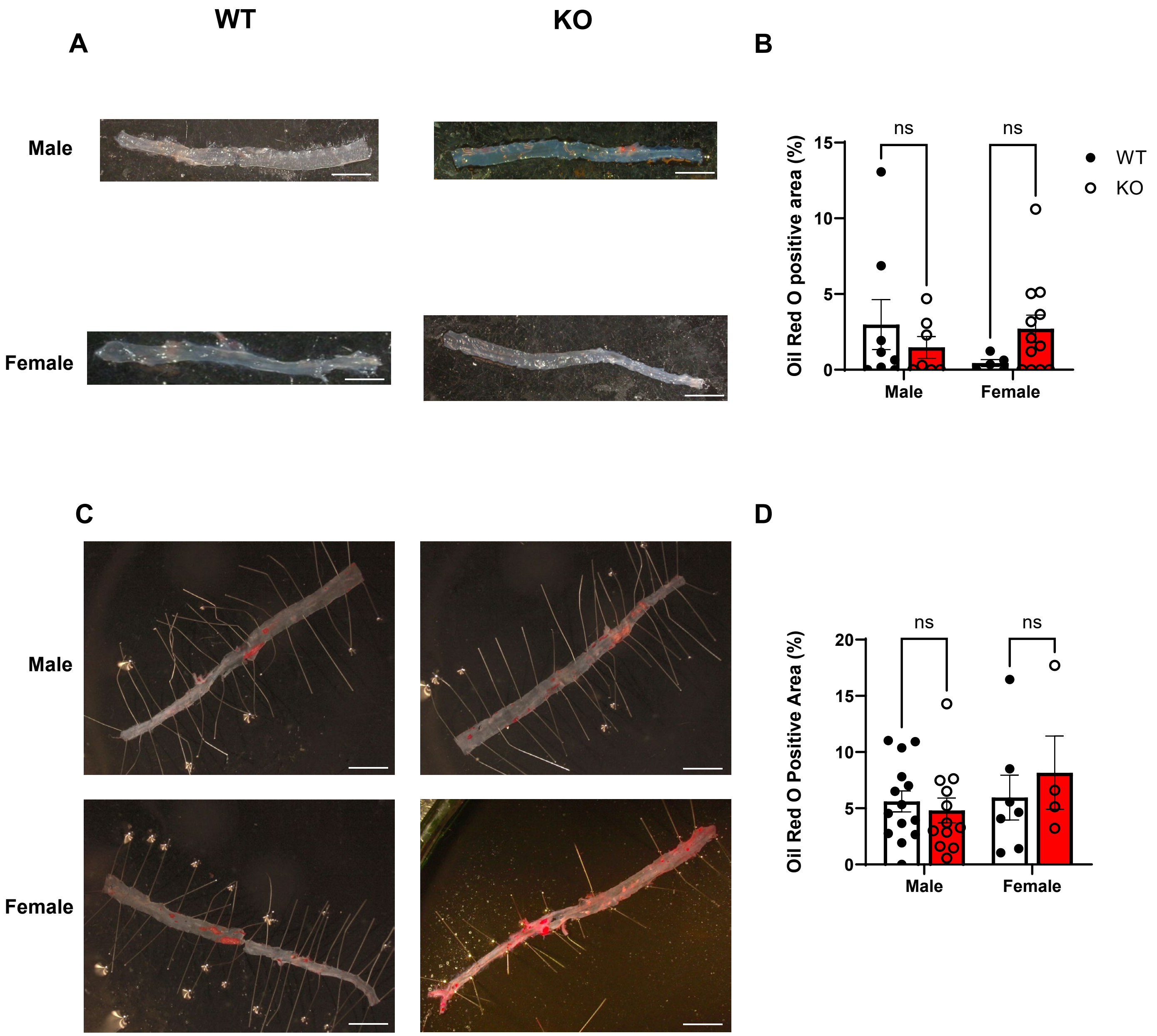

Figure S2. A, Representative images of en-face Oil-red-O-stained descending aorta after 4 weeks of HCD beginning at 6 weeks of age. Scale bar, 5 mm. B, Quantification of atherosclerotic area in the descending aorta after 4 weeks of HCD (n = 7 – 12 per genotype). C, Representative images of en-face Oil-red-O-stained descending aorta after 16 or 20 weeks of HCD starting at 6 weeks of age. D, Quantification of atherosclerotic area in the descending aorta after 16 or 20 weeks of HCD (n = 4-14 per genotype). Scale bar, 5 mm. Statistical analysis: two-way ANOVA with Sidak's multiple comparisons tests were performed. Data are presented as the mean ± SEM.

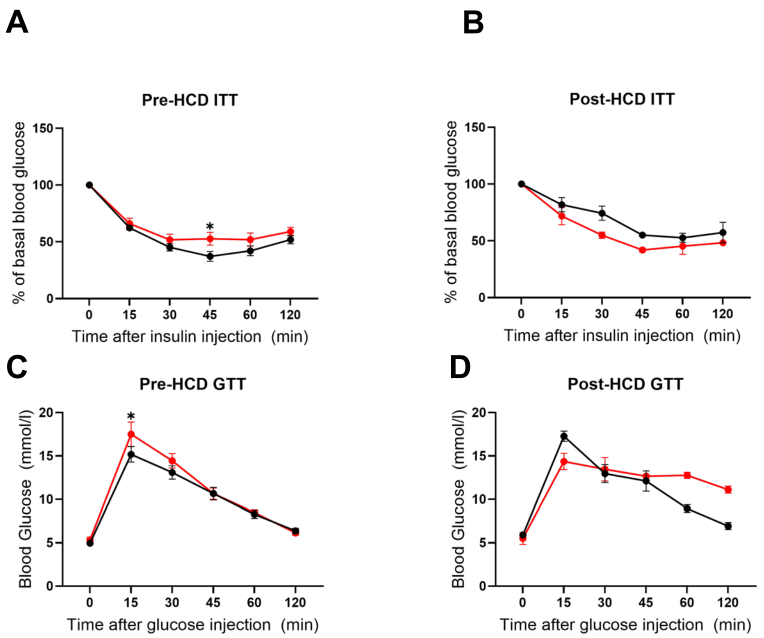

Figure S3. Metabolic parameters of WT: ApoE$^{-/-}$P-Jak2 WT and KO: ApoE$^{-/-}$P-Jak2 KO female mice. A, B, Pre- and post-HCD ITT results in female mice (n=2-15 per genotype). C, D, Pre- and post-HCD GTT results in female mice (n=2-15 per genotype). Data are presented as the mean ± SEM, Differences between groups analyzed for statistical significance by two-way ANOVA with Sidak's multiple comparisons test. *P<0.05.

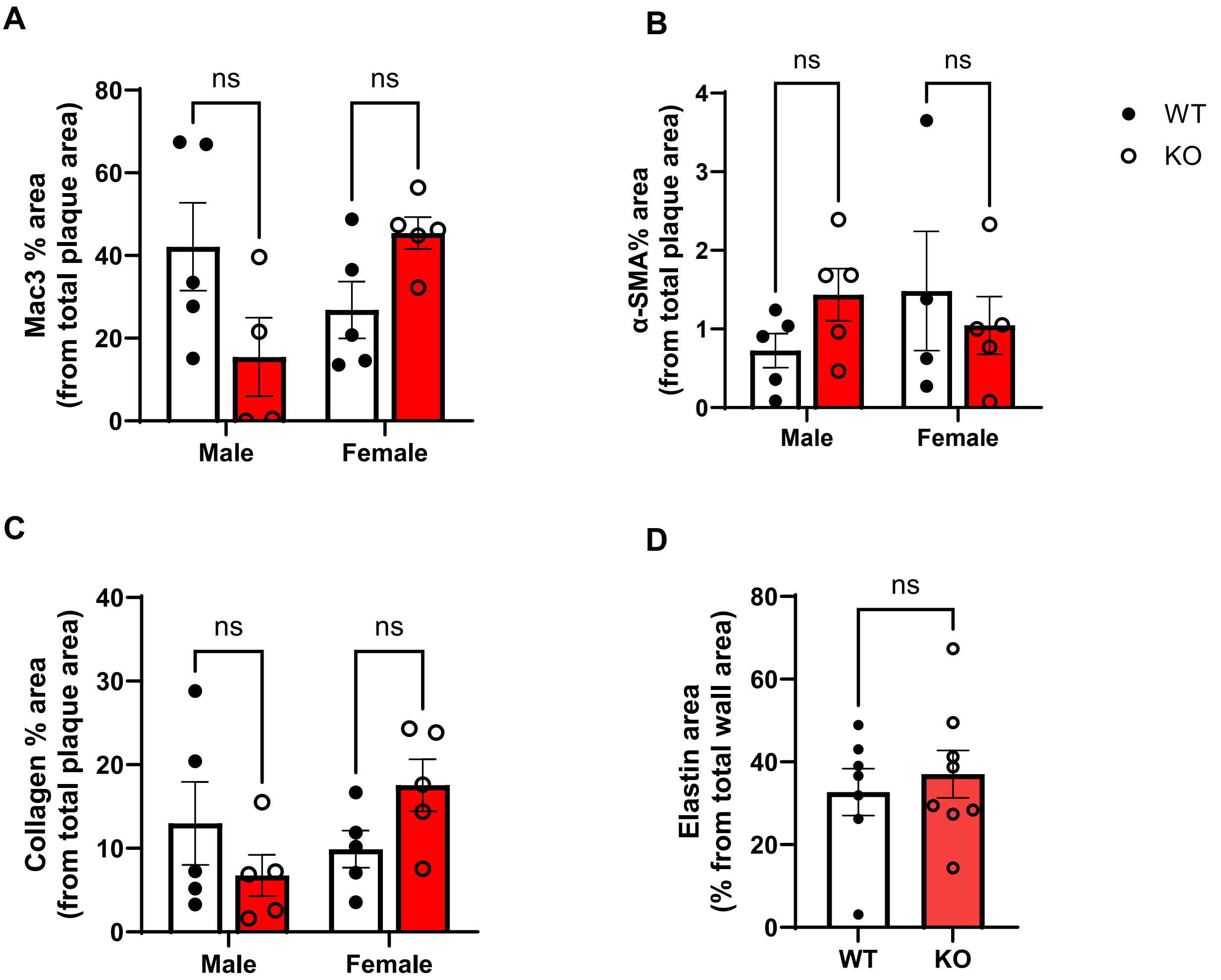

Figure S4. Plaque characterization of aortic root sections after 4 weeks of HCD. A, Quantification of Mac3 immunostained area expressed as a percentage of total plaque area separated by sex. B, Quantification of α-SMA immunostained area expressed as a percentage of total plaque area separated by sex. C, Quantification of collagen expressed as a percentage of total plaque area separated by sex. D, quantification of elastin expressed as percentage of total wall area using Masson's trichrome and Verhoeff-Van Gieson. Statistical analysis: two-way ANOVA with Sidak's multiple comparisons tests were performed for sex-separated data and two-tailed unpaired t tests were performed for elastin quantification. Data are presented as the mean ± SEM.
